# Dwell position inaccuracy in the Varian GammaMed HDR ring applicator

**DOI:** 10.1120/jacmp.v11i4.3158

**Published:** 2010-09-07

**Authors:** Robin L. Stern, Tianxiao Liu

**Affiliations:** ^1^ Department of Radiation Oncology University of California, Davis Health System Sacramento CA USA

**Keywords:** brachytherapy, high‐dose rate, HDR, tandem and ring

## Abstract

Varian has issued two Product Notification Letters warning of known inaccuracies in dwell positions for their GammaMed HDR ring applicator. This inaccuracy was measured for two sets of applicators. Autoexposed radiographs were taken of the HDR source at different dwell positions and analyzed per Varian recommendations using tools within the BrachyVision treatment planning program. Comparison between programmed and actual dwell positions showed the actual positions shifted distally by an average of 0.34 cm (0.17 cm–0.59 cm) across all positions in all rings. A correction method was developed and tested. During planning, the tip of the ring was extrapolated distally beyond its actual position in the patient image set and a proximal offset of the same distance was applied to the dwell positions. A global shift of 0.3 mm corrected all but the most proximal actual dwell position to within +2 mm of the planned position.

PACS number: 87.53Jw, 87.56Da

## I. INTRODUCTION

Tandem and ring applicators are commonly used for high‐dose‐rate (HDR) brachytherapy treatment of cervical cancer. As with all brachytherapy treatments, differences between the source positions specified during treatment planning and the actual positions during treatment can result in unintended and undesirable deviations from the planned dose distribution. In 2008, Varian issued two Product Notification Letters (PNLs) – (PNL‐VS/GM‐CR‐30271 and PNL‐GM‐CR‐30271 (2)),^(^ ^1^ ^)^ – regarding inaccuracies in dwell positions within the ring of their CT/MR Ring and Tandem Applicator due to source wire flexibility and friction within the ring channel. The resultant differences between actual and planned dwell positions can exceed the International Electrotechnical Commission (IEC) standard^(^ ^2^ ^)^ of 2 mm. The PNLs state the user needs to radiographically characterize the behavior of each ring because individual rings may vary. Furthermore, a ring's behavior may change with time, so periodic radiographic verification needs to be done. In response to these letters, characterization tests were undertaken on all three rings from two CT/MR Ring and Tandem Applicator sets. A positional correction method based on the results was developed and tested.

## II. MATERIALS AND METHODS

**Figure 1:**
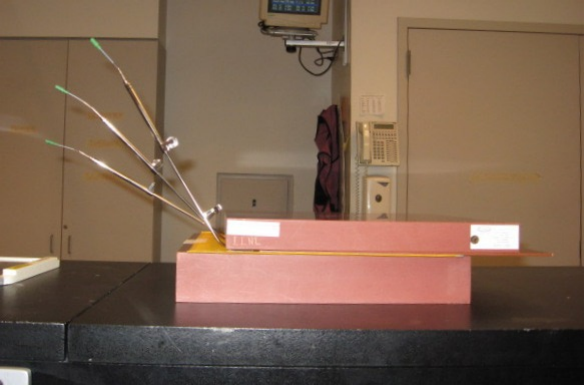
Setup used for film exposure. The three ring applicators were taped flat against a sheet of ready‐pack film then sandwiched between blocks of water‐equivalent plastic. Dummy markers are shown inserted in the rings.

All three rings (30°, 45°, and 60°) from one applicator set were taped to KODAK EDR2 Ready‐Pack film (Eastman Kodak Co., Rochester, NY) with the ring flat against the film. A 5 cm thick block of tissue‐equivalent plastic was placed under the film to provide backscatter and stabilization, with an additional 3 cm on top of the applicators, primarily to keep them in contact against the film (see Fig. 1). An X‐ray exposure was made with a conventional simulator of each individual ring either with or without a dummy marker inserted; then the applicators and plastic were moved en mass to the GammaMed*plus* HDR unit (Varian Medical Systems Inc., Palo Alto, CA) and an autoradiograph was made. Different series of programmed dwell positions were used for different films, with multiple films using the same series. Over five weeks, a total of 22 double exposures was made for the rings in Applicator Set 1 and four double exposures for the rings in Applicator Set 2. The films were digitized, converted to DICOM format, imported into Varian BrachyVision v8.2.1, and analyzed with BrachyVision software tools, per recommendations in the second PNL.

**Figure 2:**
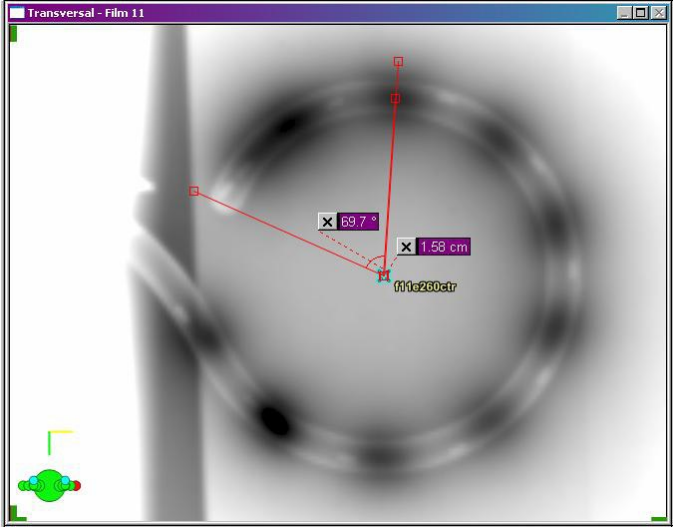
Dwell position measurement technique using BrachyVision software tools. Angle from catheter inner tip to autoradiograph exposure center and distance from ring center to autoradiograph center were measured for calculation of arc distance from catheter inner tip to autoradiograph exposure center.

Each ring image was analyzed separately. According to Varian convention, dwell positions are referenced by the distance of the source from the afterloader. Programmed dwell position 130 cm represents the furthest extension of the source and is defined with the center of the source 0.35 cm from the inner tip of the ring channel. For analysis, the autoradiograph exposures (representing the actual dwell positions) were assumed to lie along a circle. This assumption holds well for dwell positions ≥ 122 cm in the circular portion of the applicator. Dwell positions < 122 cm are in the applicator stem. The center of the ring was determined by equalizing as much as possible the distances from the ring center to the centers of all the autoradiograph exposures in the ring. The cursor, ruler, angle and window/level software tools in BrachyVision were used to first determine the center of the autoradiograph exposure circle. The angle from the inside tip of the ring channel to the center of each autoradiograph exposure was then measured, along with the radius from ring center to autoradiograph exposure center (Fig. 2). From these, arc distance was calculated to determine actual dwell position. Measured dwell positions were compared to programmed dwell positions and the average shift between them was determined. A correction method incorporating the shift was then developed for treatment planning.

Two additional films for each applicator set were exposed four months later using the planning correction method in order to both verify the method and test ring behavior stability over time and source exchanges.

## III. RESULTS

All films display an autoradiograph exposure corresponding to programmed dwell position 130 cm, since the GammaMed*plus* afterloader always extends the source fully before retracting it (if necessary) to the first programmed dwell position. This autoradiograph exposure position was found to be proximal to the nominal 130 cm position (source center 0.35 cm from ring inner tip). In addition, because of friction of the source within the ring, the first several millimeters of retraction don't move the source but only tighten the source wire against the inner radius of the ring, as described in the first PNL. Consequently, programmed dwell positions > 129.2 cm could not be resolved from the full‐extension autoradiograph exposure, and it was difficult to distinguish the autoradiograph exposure for programmed position 129.2 cm.

**Figure 3:**
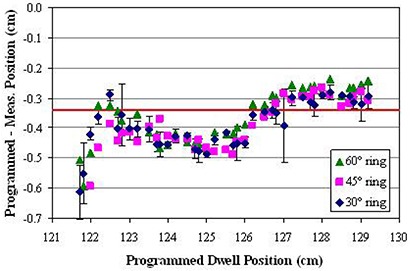
Difference between programmed and measured dwell position for all rings in Applicator Set 1. Error bars (± σ) are shown for the 30° ring. The solid line is the average difference of −0.34 cm.

**Figure 4:**
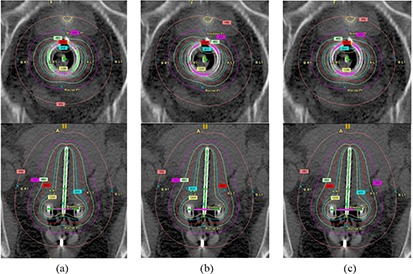
Dose distributions in and perpendicular to the plane of the ring for a representative ring‐and‐tandem treatment using a 45° ring: a) planned dwell positions; b) uncorrected actual dwell positions; c) corrected actual dwell positions.

Figure 3 shows the difference between programmed and measured dwell positions for programmed dwell positions 121.7 cm–129.2 cm for all rings in Applicator Set 1. Error bars of one standard deviation are shown only for the 30° ring for clarity; standard deviations were slightly smaller for the other two rings. Results for Applicator Set 2 are similar. In all cases, the measured position was more distal than the programmed. The average difference for all positions in all rings (both applicator sets) was −0.34 cm, ranging from −0.18 cm to −0.59 cm. There is no correlation in average difference between applicator sets or angle of rings. The effect on the calculated dose distribution for a representative patient undergoing tandem‐and‐ring treatment with a 45° ring is shown in Fig. 4. For this plan, a nominal spacing of 0.5 cm between dwell positions was used. Five positions on each lateral side of the ring were activated, as well as positions along the tandem from tip to ring. All active dwell positions were given equal dwell times, and the plan was normalized to give 600 cGy to the ICRU point A. Figure 4(a) shows the dose distribution in and perpendicular to the plane of the ring as planned, while Fig. 4(b) shows the dose as actually treated. Corresponding dose to reference and critical points are given in Table 1.

**Table 1:** Doses to a representative patient for planned, uncorrected actual, and corrected actual dwell positions.

*Point*	*Planned (Gy)*	*Uncorrected Actual (Gy)*	*Corrected Actual (Gy)*
A (right)	600.6	603.8	602.4
A (left)	599.7	587.0	597.5
B (right)	155.5	156.5	155.9
B (left)	151.4	148.1	150.6
Bladder	302.6	307.5	299.6
Rectum	247.2	252.6	250.6

### Correction method and verification

**Figure 5:**
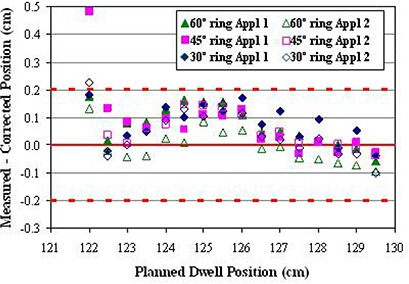
Difference between corrected planned and corresponding average measured dwell positions for all rings in both applicator sets. The dotted lines show the ± 0.2 mm IEC standard.

Dwell positions are specified within BrachyVision by designating a start position, a stop position and a fixed step size. Because of this, dwell positions cannot be corrected individually, and only a global shift/correction over the entire ring can be applied. Resolution limits this shift to integer millimeters. Furthermore, the same correction factor should be used if possible for all rings, to avoid clinical confusion and mix‐up. A shift of 0.3 cm was chosen based on the average difference between measured and programmed positions of −0.34 cm for all positions, all rings. To make the planned dwell positions as displayed on the patient image set correspond correctly to the actual delivered dwell positions, the correction method suggested in the first PNL was implemented. In planning, the defined location of the inner tip of the ring is extrapolated distally beyond its actual position by the shift correction amount of 0.3 cm, and a proximal offset of the same amount is applied to the dwell start position. This causes the dwell positions to be displayed correctly but with the associated programmed locations changed by the shift correction. The physical applicator is not affected, and the actual distance to the tip of the physical applicator remains the same. Results from the verification films show this method yields actual dwell positions that agree with the planned positions within the ± 0.2 cm IEC standard for all except the most proximal positions in two of the rings (Fig. 5). Furthermore, agreement between the verification films and the original data films shows that the behavior of the source in the rings did not change significantly over the four months and one source exchange between film sets. Figure 4(c) and Table 1 show the effect of the correction on the dose distribution for the representative patient treatment described above.

## IV. DISCUSSION

Figure 4 shows that the dwell position errors in the ring applicator due to source wire flexibility and friction cause a rotation of the dose distribution in the plane of the ring towards the tip of the ring. The effect on dose to points within the patient depends on their proximity to the ring. For this patient, the dose to the left points A and B were reduced by over 2%, while the bladder and rectal point doses were increased by 1.6% and 2.2%, respectively. Application of the correction method shifts the dose distribution back towards its planned position. Doses for left points A and B agree with the original planned values to within 0.5%, while the bladder and rectal point doses differences are reduced to 1.0% and 1.4%, respectively.

The distal direction of our measured shift is opposite the expectation of the second PNL, which states a proximal shift is more likely. The extrapolation‐and‐shift correction method will not work for a proximal shift, and Varian recommends a different method involving creating two separate plans, one which shows the desired dwell positions and dose distribution on the anatomy, and the other which gives the corrected programmed dwell positions. Although this latter method would work equally well for distal shifts, it was deemed more complex with a higher possibility of error due to confusion of the two plans.

In our data, the measured 130 cm dwell position was proximal to the expected position, unlike all the other dwell positions, so the correctional shift described above is in the wrong direction for this dwell position. In addition, the measured positions for programmed dwell positions > 129.2 can not be distinguished from the 130 cm autoradiograph exposure, while programmed dwell positions < 121.7 are in the stem of the applicator and can not be accurately measured. Therefore, only programmed dwell positions 121.7–129.2cm (corresponding to planned positions 122.0–129.5cm) are allowed for patient treatment. This has minimal clinical affect, since the disallowed positions are rarely wanted.

## V. CONCLUSIONS

Actual source dwell positions were radiographically measured for two sets of Varian GammaMed HDR ring applicators and found to be shifted distally an average of 0.34 cm from the programmed positions. A correction method involving extrapolation of the applicator tip and a start position offset during planning was implemented and tested. A single shift value of 0.3 cm for all rings yielded acceptable agreement between planned and delivered source positions for all rings.
